# Association of daily step count and serum testosterone among men in the United States

**DOI:** 10.1007/s12020-021-02631-2

**Published:** 2021-02-12

**Authors:** Francesco Del Giudice, Frank Glover, Federico Belladelli, Ettore De Berardinis, Alessandro Sciarra, Stefano Salciccia, Alex M. Kasman, Tony Chen, Michael L. Eisenberg

**Affiliations:** 1 Department of Maternal-Infant and Urological Sciences, “Sapienza” University or Rome, Policlinico Umberto I Hospital, Rome, Italy; 2 Department of Urology, Stanford University School of Medicine, Stanford, CA USA; 3 Emory School of Medicine, Emory University, Atlanta, GA USA; 4 Division of Experimental Oncology/Unit of Urology, University Vita-Salute San Raffaele, IRCCS Hospital San Raffaele, Milan, Italy; 5 Department of Obstetrics and Gynecology, Stanford University School of Medicine, Stanford, CA USA

**Keywords:** Male General Health, Physical activity, Steps count, Testosterone level, NHANES, Accelerometer data

## Abstract

**Purpose:**

To describe the association between daily activity (i.e., daily step counts and accelerometer intensity measures) and serum TT levels in a representative sample of US adults aged 18 years or older.

**Methods:**

A retrospective cohort study was carried out utilizing the NHANES (National Health and Nutrition Examination Survey) 2003–2004 cycle. Physical activity was measured with a waist-worn uniaxial accelerometer (AM-7164; ActiGraph) for up to 7 days using a standardized protocol. Using linear and multivariable logistic regression controlling for relevant social, demographic, lifestyle, and comorbidity characteristics, we assessed the association between daily step counts and TT.

**Results:**

A total of 279 subjects with a median age 46 (IQR: 33–56) were included in the analysis. 23.3% of the cohort had a low serum TT level (TT < 350 ng/dl). Compared to men who took <4000 steps per day, men who took >4000 or >8000 steps/day had a lower odd of being hypogonadal (OR 0.14, 95% CI: 0.07–0.49 and 0.08, 95%CI: 0.02–0.44, respectively). While a threshold effect was noted on average, TT increased 7 ng/dL for each additional 1000 steps taken daily (β-estimate: 0.007, 95% CI: 0.002–0.013).

**Conclusions:**

Patients with the lowest daily step counts had higher odds of being hypogonadal. The current work supports a possible association between daily steps, total testosterone, and hypogonadism for men in the US.

## Introduction

Testosterone is necessary for normal male development and function. Abnormal testosterone levels have been associated with changes in male body muscle mass and fat distribution as well as bone metabolism and energy levels [1]. Male hypogonadism comprises of both persistent-specific symptoms and biochemical evidence of testosterone deficiency [2]. Hypogonadism becomes more prevalent with age. In particular, the EMAS study reported a 0.4% per annum decrease in total testosterone (TT) and a 1.3% per annum decline in free testosterone (fT) [3]. In addition, there is a high prevalence of hypogonadism within specific populations, including patients with type 2 diabetes, metabolic syndrome, obesity, and low performance status [4, 5].

Given the association between serum testosterone and health, it is reasonable to assume that physical activity (PA) is also associated with TT [6]. PA is an important source of physical, psychological, cognitive, and social health benefit for all age groups, including the prevention of muscle-skeletal fragility events, which may ultimately lead to long-term pain, loss of function, and higher mortality rates in the elderly [7]. This relationship is evident in prostate cancer (PCa) survivors treated with androgen deprivation therapy (ADT) who are at higher risk for muscle mass and muscular strength decrease, loss of bone density as well as increasing in body weight and fat mass [8]. However, previous studies from the National Health and Nutrition Examination Survey (NHANES) [9, 10] were unable to detect any association between overall circulating TT levels and the amount of PA as assessed by survey responses. However, self-reports of type, duration, and intensity of PA, may be affected by recall and social desirability bias as well as by individual perception of PA intensity [11, 12]. Objective measures of PA through activity monitors may provide more reliable measures. The purpose of the current study was to describe the association between daily activity (i.e., accelerometer intensity measures) and serum TT, fT, and Bioavailable T levels in a representative sample of US adults aged 18 years or older.

## Materials and methods

### Study population

Data from the NHANES cycle 2003–2004 were analyzed (available from: https://wwwn.cdc.gov/nchs/nhanes/2003-2004/PAXRAW_C.htm). NHANES consists of a noninstitutionalized US civilians sample, using a multistage probability sampling design that considers geographical area and minority representation via a cross-sectional survey conducted by the National Center for Health Statistics (NCHS) of the Centers for Disease Control and Prevention (available from https://wwwn.cdc.gov/nchs/nhanes/analyticguidelines.aspx). Sample weights are generated to create nationally representative estimates for the US population and subgroups defined by age, sex, and race/ethnicity [13]. As the analysis utilized deidentified data with no direct participant contact, it is not considered to be human subjects research and consequently does not require institutional review board approval.

Demographic information (i.e., age, ethnicity, education), health behaviors (smoking, BMI) and concomitant comorbidities were also collected. The main inclusion criteria were participants who were at least 18 years or older with available serum samples in the repository and also wore an ActiGraph model 7164 accelerometer on the hip during waking hours for a 7-day period with at least 1 day of valid wear (i.e., ≥10 h/d).

Following an overnight fast, men serum samples were firstly dawned between 8.30 and 11.30 a.m. and then testosterone concentrations were determined using a competitive electrochemiluminescence immunoassay on the 2010 Elecsys autoanalyzer (Roche Diagnostics, Indianapolis, IN, USA) with the lowest detection limit of the assay being 0.02 ng/mL. All sex steroid hormones from the present NHANES cycle were assayed at Boston Children’s Hospital (Boston, MA, USA) by laboratory technicians blinded to participant characteristics. The details for the NHANES laboratory methodology for testosterone determination are available from: https://wwwn.cdc.gov/nchs/nhanes/2003-2004/SSCHL_C.htm. fT and Bioavailable T were then separately calculated given the serum values of sex-hormone binding globulin and albumin levels in accordance with the formula described by Vermeulen et al. [14], available from http://www.issam.ch/freetesto.htm.

Men were excluded from the analysis if they were diagnosed with medical history of PCa (as they may be treated with hormone ablation therapy), reported limitations to engaging in PA (i.e., unable to walk without an assistive device), or were missing information on testosterone/accelerometer data or on covariates of interest. Of 2922 men aged at least 18 years old at the time of the survey, 2052 had valid accelerometer data. A total of 386 of the 2052 with accelerometer data also presented with available serum TT information. Out of these, 107 (27.7%) were excluded due to missing data reaching a final sample size of 279 subjects enrolled into analysis.

### Accelerometer-measured PA

PA was measured with a waist-worn uniaxial accelerometer (AM-7164; ActiGraph LLC; Ft. Walton Beach, FL) for up to 7 days using a standardized protocol [13] within the same data acquisition time frame for each NHANES participant. The signals are then filtered and digitized by converters in the device and summed over a user-specified period of time (epoch) to provide activity counts per epoch, commonly expressed as count per minute or per day (CPD). Data were initially screened for non-wear time using a previously developed algorithm for NHANES accelerometer data [13]. Days with fewer than 10 h of wear time were excluded and participants with at least 1 valid day of accelerometer data were included in the analysis.

### Pax intensity assessment

The physical activity monitors used in NHANES collected objective information on the intensity and duration of common locomotion activities such as walking, and jogging defined by the “*PAXINTEN*” variable, which correspond to the sequential observation number in minutes as recorded by the monitor device intensity value (available from: https://wwwn.cdc.gov/nchs/nhanes/2003-2004/PAXRAW_C.htm). Each day of wear produces 1440 individual minute records up to the last minute of day 7. Pax intensity values were classified into weighted quartiles and modeled upon TT serum levels and the available covariates information. The 25th percentile (~<155078.6 CPD) was used as the reference [13].

### Multiple imputation method for daily steps count determination

Although activity count data from the 2003–2004 NHANES cycle has been publicly available, step count data from this cycle were not released because of missing data. Several research teams previously addressed this issue by utilizing a semi-parametric multiple imputation method, whereby bootstrapping and ordinary least squares regression provided accurate values for missing data [15, 16]. This imputation method uses demographic, questionnaire, laboratory and accelerometer data on participants who are not missing steps values, to predict step values for individuals who are missing daily step values. Prior literature has validated the imputation [15]. Ordinary least squares regression on newly imputed dataset was compared to those of the pre-imputation dataset, and similar estimates of linear and/or logistic regression analysis were found between both datasets.

### Statistical analysis

Following the recommended guidelines from the NCHS (Centers for Disease Control and Prevention 2012a), all the analyses were performed with appropriate weights for the complex survey sampling method of NHANES data. Student’s *t* test and chi-squared statistic were used to assess differences among means and proportions between continuous variables and subgroups, respectively. To explore the relationship concerning step CPD or pax intensity CPD, we examined the odds of impaired TT levels (i.e., <350 ng/dl), fT (<6.5 ng/dl), and bioavailable T (<110 ng/dl) in jointly classified categories with low step counts (<4000 steps/day) and low pax intensity (lower quartile) as reference group. Linear regression was used to estimate and compare variation of serum TT concentrations across cumulative recorded steps and pax intensity measures. One-way ANOVA on ranks (Kruskal–Wallis test) was used to test the differences concerning continuous TT levels between groups categories (steps categories and pax intensity quartiles). Multivariable logistic regression was used to estimate the odds of low TT, fT, and Bioavailable T for varying step and pax intensity levels. Sensitivity analyses were performed using different TT thresholds and associations with step counts. The TT threshold was chosen from established values where men are recommended to consider hormone replacement therapy by several International American/European Andrological and/or Endocrinological Societies and similar to prior analyses [10]. Locally weighted scatter-plot smoother (LOWESS) function was used to graphically depict the relationship between continuous step/pax intensity counts and TT, fT, and Bioavailable T. The analysis was adjusted for covariates selected based on previous investigation, including age, BMI, race/ethnicity (non-Hispanic white, Mexican American, non-Hispanic black), education level (less than high school, high school, greater than high school), and smoking status (current vs. never). Moreover, the model was adjusted for all comorbidity covariates known to influence both TT homeostasis (alcohol consumption, diabetes, hypercholesterolemia, hypertension, cancer) as well as participant’s motility limitation (stroke, coronary artery disease, heart failure). Data analysis was performed using SAS v.9.2 (Cary, NC, USA) with *p* values < 0.05 considered statistically significant.

## Results

279 men had complete data regarding accelerometer, serum TT, and covariates of interest. Overall, 214 (76.7%) men had normal TT levels and 65 men had low TT levels. Men presenting with normal TT levels (i.e., >350 ng/dl; mean 581.1 ± 182 vs. 248 ± 75) were significantly younger (*p* = 0.045) and had a lower mean BMI (*p* = 0.006) compared to men with lower TT levels. No significant nor clinically relevant differences were identified with regard to other socio-demographic, or comorbidities distribution (Table 1). Participants took a mean of 8702.2 (SD: 4337.2) steps per day while the mean pax intensity value was 249,848.5 (SD: 139,941.1). TT, fT, and Bioavailable T levels were consistently different across the step categories (*p* = 0.012, 0.001, and 0.005, respectively) and pax intensity quartiles (*p* = 0.026, 0.000, and 0.146, respectively) (Table 2).

**Table 1:** Socio-demographic and clinical characteristics of the 279 subjects enrolled from the NHANES 2003–2004 cycle according to normal vs. low total testosterone distribution (ng/dl)

Variables		Total testosterone among NHANES participants		
		>350 ng/dl	<350 ng/dl	*p* value
Sample size, *n* (%)		214 (76.7)	65 (23.3)	–
Total testosterone, ng/dl	Mean (SD)	562.6 (168.3)	247.4 (75.3)	0.001
	Median (IQR)	520 (426–649)	268 (191–314)	
Free testosterone, ng/dl	Mean (SD)	25.1 (16.3)	11.7 (7.1)	0.004
	Median (IQR)	18.3 (11.2–29.6)	8.5 (5.5–14–3)	
Bioavailable testosterone, ng/dl	Mean (SD)	171.8 (109.4)	82.8 (51.6)	0.001
	Median (IQR)	128 (67–246.5)	64.7 (30.3–95.3)	
Age, years	Mean (SD)	44.1 (15.3)	50.3 (16.7)	0.045
	Median (IQR)	44 (32–53)	54 (35–63)	
Age, group class, *n* (%)	18–39	80 (37.4)	13 (20)	0.11
	40–59	69 (32.2)	19 (29.2)	
	60+	65 (30.4)	33 (50.8)	
	Mexican American	43 (20.1)	12 (18.5)	
Race/ethnicity, *n* (%)	Non-Hispanic black	42 (19.6)	10 (15.4)	0.37
	Non-Hispanic white	129 (60.3)	43 (66.2)	
BMI	Mean (SD)	27.1 (4.7)	31.3 (5.9)	0.006
	Median (IQR)	26.5 (24.2–30.1)	30.7 (28–35)	
BMI group class, *n* (%)	18.5–25	71 (33.2)	8 (12.3)	0.037
	25–30	87 (40.7)	20 (30.8)	
	>30	56 (26.2)	37 (56.9)	
Education group class, *n* (%)	<High school	62 (29)	19 (29.2)	0.31
	High school	57 (26.6)	13 (20)	
	>High school	95 (44.4)	33 (50.8)	
Smoking status, *n* (%)	Never smoker	88 (41.1)	34 (52.3)	0.27
	Active smoker	126 (58.9)	31 (47.7)	
Comorbidities				
Alcohol consumption, *n* (%)	≤2 Drink/day	99 (46.3)	39 (60)	0.08
	>2 Drink/day	115 (53.7)	26 (40)	
Diabetes, *n* (%)	No	195 (91.1)	50 (76.9)	0.41
	Yes	19 (8.9)	15 (23.1)	
High cholesterol, *n* (%)	No	193 (90.2)	50 (76.9)	0.16
	Yes	21 (9.8)	15 (23.1)	
Hypertension, *n* (%)	No	179 (83.6)	47 (72.3)	0.21
	Yes	35 (16.4)	18 (27.7)	
Stroke, *n* (%)	No	207 (96.7)	60 (92.3)	0.004
	Yes	7 (3.3)	5 (7.7)	
Coronary artery disease, *n* (%)	No	205 (95.8)	61 (93.8)	0.57
	Yes	9 (4.2)	4 (6.2)	
Heart failure, *n* (%)	No	209 (97.7)	62 (95.4)	0.61
	Yes	5 (2.3)	3 (4.6)	
Cancer, *n* (%)	No	195 (91.1)	61 (93.8)	0.03
	Yes	19 (8.9)	4 (6.2)

**Table 2:** Steps and pax intensity count per day (CPT) according to normal, normal-low, and low total testosterone values within the study population

Variables		Normal total testosterone	Normal-low total testosterone	Low total testosterone		Normal free testosterone	Low free testosterone		Normal bioavailable testosterone	Low bioavailable testosterone	
		>350 ng/dl, (*n* = 214)	<350 and >250 ng/dl, (*n* = 37)	<250 ng/dl, (*n* = 28)	*p* value	>6.5 ng/dl (*n* = 231)	<6.5 ng/dl, (*n* = 41)	*p* value	>110 ng/dl, (*n* = 128)	<110 ng/dl (*n* = 145)	*p* value
Steps count, (CPD) *n* (%)	<4000	19 (8.9)	11 (29.7)	9 (32.1)	0.012	22 (9.5)	13 (31.7)	0.001	16 (12.5)	19 (13.1)	0.005
	4000–8000	84 (39.3)	15 (40.5)	12 (42.9)		91 (39.4)	19 (46.3)		56 (43.8)	55 (37.9)	
	8000–12000	74 (34.6)	5 (13.5)	4 (14.3)		74 (32)	8 (19.5)		40 (31.3)	42 (29)	
	>12000	37 (17.3)	6 (16.2)	3 (10.7)		44 (19)	1 (2.4)		16 (12.5)	29 (20	
Pax intensity, (CPD) *n* (%)	1st Quartile	53 (24.8)	15 (40.5)	15 (53.6)	0.026	48 (20.8)	30 (73.2)	0.000	37 (18)	42 (29)	0.148
	2nd Quartile	56 (26.2)	11 (29.7)	8 (28.6)		69 (29.9)	5 (12.2)		41 (21.1)	33 (22.8)	
	3rd Quartile	56 (26.2)	4 (10.8)	3 (10.7)		58 (25.1)	4 (9.8)		27 (32)	35 (24.1)	
	4th Quartile	49 (22.9)	7 (18.9)	2 (7.1)		56 (24.2)	2 (4.9)		23 (28.9)	35 (24.1)	
Steps count, (CPD)	Mean (SD)	9040.7 (4139.9)	7453.7 (4,529.2)	7681.2 (5,044.6)	0.36	8713.1 (4178.3)	5491.2 (2928.5)	0.001	7869.9 (3819.1)	8525.4 (4438.4)	0.063
	Median (IQR)	8968 (6187–11,364)	6401 (3714–10,658)	5919 (3342–10,530)		8145.9 (5744.2–11145.3)	5392.9 (2778.0–7019.3)		7197.6 (5397.9–9747.4)	7877.1 (5346.4–11,268.9)	
Pax intensity, (CPD)	Mean (SD)	260,062.3 (135,858.5)	223,586 (142,369.8)	205,381.9 (153,465.4)	0.48	254,275 (134,872)	126,289.2 (88,548.3)	0.000	220,091.4 (129,935.3)	246,889.8 (141,813.7)	0.067
	Median (IQR)	261,770 (172,900–334,775)	220,285 (101,542–262,567)	167,623 (132,845–329,726)		235,733.7 (163,176.4–329,304.9)	94,982.3 (67,893.9–154,648.6)		201,194.7 (131,909.9–288,193.8)	233,415.7 (142,522.9–328,884.1)	

### Daily step count and TT levels

**Fig. 1:**
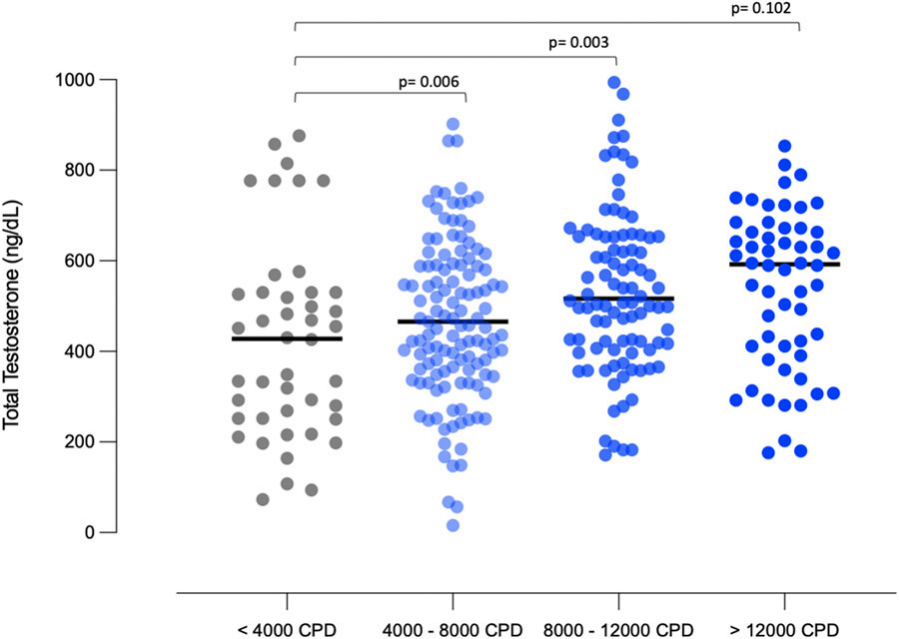
Multivariable adjusted one-way ANOVA on ranks assessing differences concerning continuous total testosterone levels between steps groups (count per day [CPD]) categories

**Fig. 2:**
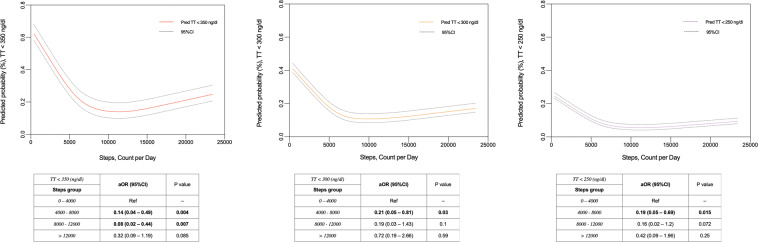
Locally weighted scatter-plot smoother (LOWESS) function depicting multivariable adjusted predicted probability of impaired total testosterone (TT, ng/dl) according to total steps (count per day [CPD])

**Fig. 3:**
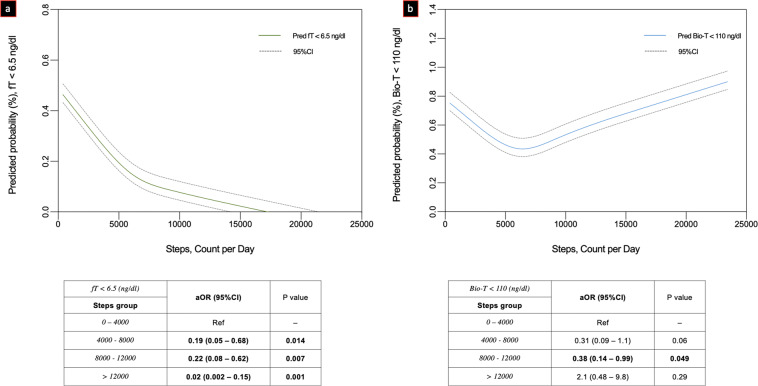
Locally weighted scatter-plot smoother (LOWESS) function depicting multivariable adjusted predicted probability of impaired free (**a**) and bioavailable (**b**) testosterone (fT, Bio-T, ng/dl) according to total steps (count per day [CPD])

When examining the association between low testosterone levels and daily step count, the odds for impaired TT was lower with increasing step count (Fig. 1). Compared to taking <4000 steps per day, men who took 4000–8000 or 8000–12,000 had a lower odd of hypogonadal TT levels (OR 0.14, 95% CI: 0.07–0.49 and 0.08, 95% CI: 0.02–0.44, respectively). While there was a threshold effect, there was a positive association with daily step count and TT levels (β-estimate: 0.007, SE: SE: 0.004, 95% CI: 0.002–0.013). For each 1000 steps, TT levels increase by 7 ng/dl. In addition, when examining steps as a continuous variable, the odds of hypogonadism also fell with each additional step (Fig. 2). Sensitivity analyses defining hypogonadism as TT < 300 and <250 ng/dl showed similar point estimates and trend toward significance (OR_300_: 0.21, 95% CI: 0.05–0.81 and 0.19, 95% CI 0.03–1.43; OR_250_: 0.19, 95% CI: 0.05–0.69 and 0.16, 95% CI: 0.02–1.2, for 4000–8000 and 8000–12,000 CPD, respectively) (Fig. 2). Higher threshold categories for steps and pax intensity (i.e., >12,000) were also examined (Fig. 2). Similarly, the adjusted odd for both impaired fT and Bioavailable T was confirmed to be significantly lower as going through the steps thresholds categories (Fig. 3a, b, respectively).

### Daily pax intensity and TT levels

Similar findings to the daily step count data were confirmed when assessing the association between pax intensity and TT levels (Supplementary Fig. 1). A positive association between TT levels and pax intensity was seen (*p* = 0.001). On multivariable logistic regression, we confirmed a lower odd of hypogonadism, fT, and Bioavailable T with increasing pax intensity levels. LOWESS function showed analogous trajectory revealing the reduced probability of hypogonadism as well as fT and Bioavailable T with cumulative increase of pax intensity values per day (Supplementary Figs. 2 and 3a, b, respectively).

## Discussion

In the present cross-sectional survey, we observed a positive association between daily step count and TT levels. Moreover, as daily steps increase, the odds of hypogonadism declines across a range of serum TT hypogonadism thresholds. Men with more than 4000 steps per day had a significantly lower odds of having low TT levels. To our knowledge, this is the first study to report an association between daily step count and serum testosterone levels.

The interaction between various measures of PA and testosterone has been studied. Previous experiences have addressed this relationship relying on self-reports on PA intensity and/or overall duration. Muller et al. [17] demonstrated that greater TT levels were associated with higher PA in subjects aged 40–80 years old. However, PA was assessed in the year prior to the survey susceptible to recall bias. Evidence suggests that the increase in circulating testosterone levels only occurs within a short period of time from the onset of exercise and returns to baseline thereafter [18]. Moreover, the acute exercise-induced boost in TT has a smaller magnitude of increase and relatively shorter time benefit [19].

While self-report is the most cost-effective and simple method to measure PA [11, 20] and can provide estimates of the type, duration, and exercise intensity in population-based studies; the differing questionnaires adopted and activity definitions often make it difficult to compare studies. For example, a cross-sectional analysis on 696 men from the European Prospective Investigation into Cancer and Nutrition study reported that high levels of vigorous exercise (i.e., 3 or more hours/week) were associated with an 11% higher testosterone concentration compared with those who reported no vigorous exercise [21]. However, no association was observed between total recreational exercise-time (<7.5, 7.5–14, or >14 h per week) and serum TT levels. In contrast, two studies examined the NHANES dataset with regard of sex steroid hormone levels and PA [10, 22]. The authors analyzed that NHANES cycles analyzed were the 1988–1991, 1991–1994, and 1999–2004 and reported that men in the highest category of frequency of PA had higher concentrations of TT consistent with the current report. Of note, Shiels et al. [22] found that those participants who had the highest PA per week had higher mean levels of TT though participating in vigorous exercise was not associated with higher TT levels. In contrast, Steeves et al. [10] noted an association between PA and TT only in those expending the highest level of energy and who were non-obese.

The etiology is uncertain, but some investigators have hypothesized mechanisms of action involving increasing caloric expenditure [23, 24]. For example, in vitro and in vivo evidence suggests a possible role for both the HPA axis as well as direct effect of exercise on the regulation of body mass composition. The effect of physical exercise in a rabbit model with metabolic syndrome-associated hypogonadotropic hypogonadism showed a down regulation of the majority of steroidogenic enzymes leading to T synthesis. Interestingly, the increase in genes related to inflammation, estrogen signaling, and glucose metabolism observed in metabolic syndrome were significantly reduced after the rabbits were exercise-trained to run on a treadmill for a 12-week period. In this model, Corona et al. [24] demonstrated how the expression of Kiss 1 gene and its receptor (Kiss1R) (neurotransmitters regulating GnRH secretion) functions, decreased orexigenic and GnRH-inhibiting factors (dynorphin and its receptors OPRD1 and OPRK1), as well as increased anorexigenic ones (proopiomelanocortin) were significantly restored in the group of exercise-trained cavies.

Furthermore, studies from human professional sports have historically shown how physical effort directly damage muscle fibers leading to a subsequent increase in concentration of anabolic hormones such as insulin-growth factor-1 (IGF-I) or growth hormone (GH) [25]. Moreover, physical expenditure has been found to significantly change the Cortisol/fT ratio that can impact on the secretion of GH and IGF-I from liver and finally on the fat-free body mass composition by blocking E_2_-mediated negative feedback on the HPA axis, thus increasing luteinizing (LH) secretion and ultimately testosterone production [26]. This can be also empirically observed in the healthy hypogonadal subjects and/or individuals with testosterone deficiency (ADT for PCa or administration of GnRH agonists) who have been presenting overall lower muscle volume, strength and function, higher fat mass, and higher incidence of insulin resistance, when compared to age-matched eugonadal men [27, 28]. In addition, there is a relevant body of evidence suggesting that testosterone therapy preserves muscle strength and power in aging men. Magnussen et al. [29] found that testosterone administration improved muscle mechanical and physical function in addition to increasing lean leg mass and total lean body mass in men aged 50–70 years with type 2 diabetes and Bioavailable T levels <7.3 nmol/L thus corroborating the importance of androgen homeostasis in men’s health.

Certain limitations warrant mention. First, only a limited number of NHANES respondents had available data for analysis. Nevertheless, we did identify a significant association between daily step count and TT. Importantly, the NHANES data reported in the present study must be considered observational and no causal inferences may be deemed. Moreover, the information concerning the total steps count were extrapolated through multiple imputation inference strategy given that original information was not released due to missing data in this specific 2003/04 cycle. However, the same techniques have previously been reported by Saint-Maurice et al. in their analysis of daily steps count and overall mortality within NHANES [16]. In addition, TT samples were not measured with liquid chromatography tandem mass spectrometry, which is considered the gold standard testosterone determination while moreover, no sufficient data were available on medications assumed by single participants thus precluding the possibility to further adjust our model on specific drugs such as morphine or oral glucocorticoids. Finally, we were unable to determine each man’s “valid wearing days” for the accelerometer nor extrapolate the relative influence of sport activities on the overall step CPD, which may limit the interpretation. The relative influence of those participants with only 1 or 2 “valid wearing days” or those actively involved in sports activities might indeed possibly model the contribution of the accelerometer-measured daily step count by over- or underestimating the overall effect sizes described in the present study.

## Conclusion

Our present analysis is the first study assessing PA volume using objective measures of PA. We found that a higher physical expenditure quantified by the daily step count (or pax intensity) taken by each individual was associated with a lower odds of hypogonadal ranges total, free, and bioavailable T levels. While there were differences in baseline BMI and age among men with and without hypogonadism, the current work supports an association between daily steps, serum testosterone levels, and the risk of hypogonadism.

## Supplementary information

Supplementary Figures 1

## References

[1] Kelly DM, Jones TH (2013). Testosterone: a metabolic hormone in health and disease. J. Endocrinol..

[2] Salonia A, Rastrelli G, Hackett G (2019). Paediatric and adult-onset male hypogonadism. Nat Rev Dis Primers.

[3] Wu FC, Tajar A, Pye SR (2008). Hypothalamic-pituitary-testicular axis disruptions in older men are differentially linked to age and modifiable risk factors: the European Male Aging Study. J. Clin. Endocrinol. Metab..

[4] Kapoor D, Aldred H, Clark S, Channer KS, Jones TH (2007). Clinical and biochemical assessment of hypogonadism in men with type 2 diabetes: correlations with bioavailable testosterone and visceral adiposity. Diabetes Care.

[5] Ding EL, Song Y, Malik VS, Liu S (2006). Sex differences of endogenous sex hormones and risk of type 2 diabetes: a systematic review and meta-analysis. JAMA.

[6] Wang C, Nieschlag E, Swerdloff RS (2009). ISA, ISSAM, EAU, EAA and ASA recommendations: investigation, treatment and monitoring of late-onset hypogonadism in males. Aging Male.

[7] Rhodes RE, Janssen I, Bredin SSD, Warburton DER, Bauman A (2017). Physical activity: health impact, prevalence, correlates and interventions. Psychol Health.

[8] Thorsen L, Courneya KS, Stevinson C, Fosså SD (2008). A systematic review of physical activity in prostate cancer survivors: outcomes, prevalence, and determinants. Support Care Cancer.

[9] Shiels MS, Rohrmann S, Menke A (2009). Association of cigarette smoking, alcohol consumption, and physical activity with sex steroid hormone levels in US men. Cancer Causes Control.

[10] Steeves JA, Fitzhugh EC, Bradwin G, McGlynn KA, Platz EA, Joshu CE (2016). Cross-sectional association between physical activity and serum testosterone levels in US men: results from NHANES 1999–2004. Andrology.

[11] Dishman RK, Richard AW, Dale AS (2001). Measurement of physical activity. Quest.

[12] Hallal PC, Reichert FF, Clark VL (2013). Energy expenditure compared to physical activity measured by accelerometry and self-report in adolescents: a validation study. PloS ONE.

[13] Troiano RP, Berrigan D, Dodd KW, Mâsse LC, Tilert T, McDowell M (2008). Physical activity in the United States measured by accelerometer. Med. Sci. Sports Exerc..

[14] Vermeulen (1999). A critical evaluation of simple methods for the estimation of free testosterone in serum. J. Clin. Endocrinol. Metab..

[15] Liu B, Yu M, Graubard BI, Troiano RP, Schenker N (2016). Multiple imputation of completely missing repeated measures data within person from a complex sample: application to accelerometer data in the National Health and Nutrition Examination Survey. Stat. Med..

[16] Saint-Maurice PF, Troiano RP, Bassett DR (2020). Association of daily step count and step intensity with mortality among US adults. JAMA.

[17] Muller M, den Tonkelaar I, Thijssen JH, Grobbee DE, van der Schouw YT (2003). Endogenous sex hormones in men aged 40–80 years. Eur. J. Endocrinol..

[18] Swerdloff RS, Wang C (1993). Androgens and aging in men. Exp. Gerontol..

[19] Zmuda JM, Thompson PD, Winters SJ (1996). Exercise increases serum testosterone and sex hormone-binding globulin levels in older men. Metabolism.

[20] Warren JM, Ekelund U, Besson H, Mezzani A, Geladas N, Vanhees L (2010). Assessment of physical activity—a review of methodologies with reference to epidemiological research: a report of the exercise physiology section of the European Association of Cardiovascular Prevention and Rehabilitation. Eur. J. Cardiovasc. Prev. Rehabil..

[21] Allen NE, Appleby PN, Davey GK, Key TJ (2002). Lifestyle and nutritional determinants of bioavailable androgens and related hormones in British men. Cancer Causes Control.

[22] Shiels MS, Rohrmann S, Menke A (2009). Association of cigarette smoking, alcohol consumption, and physical activity with sex steroid hormone levels in US men. Cancer Causes Control.

[23] Corona G, Rastrelli G, Monami M (2013). Body weight loss reverts obesity-associated hypogonadotropic hypogonadism: a systematic review and meta-analysis. Eur. J. Endocrinol..

[24] Corona G, Rastrelli G, Morelli A (2020). Treatment of functional hypogonadism besides pharmacological substitution. World J. Mens Health.

[25] M. Barbara, K. Anna, Z.L. Agnieszka, The impact of professional sports activity on GH-IGF-I axis in relation to testosterone level. Am. J. Mens Health **14**(1) (2020). 10.1177/1557988319900829 10.1177/1557988319900829 PMC7047237 32102608

[26] Del Giudice F, Busetto GM, De Berardinis E (2020). A systematic review and meta-analysis of clinical trials implementing aromatase inhibitors to treat male infertility. Asian J. Androl..

[27] Bhasin S, Storer TW, Singh AB, Woodhouse L, Singh R, Artaza J (2004). Testosterone effects on the skeletal muscle. Testosterone: Action, Deficiency, Substitution.

[28] Gonzalez BD, Jim HS, Small BJ, Sutton SK, Fishman MN, Zachariah B (2016). Changes in physical functioning and muscle strength in men receiving androgen deprivation therapy for prostate cancer: a controlled comparison. Support. Care Cancer.

[29] Magnussen LV, Hvid LG, Hermann AP, Hougaard DM, Gram B, Caserotti P, Andersen MS (2017). Testosterone therapy preserves muscle strength and power in aging men with type 2 diabetes-a randomized controlled trial. Andrology.

