# Supplementary material for: Association of daily step count and serum testosterone among men in the United States
Source: Endocrine. 2021 Feb 12;72(3):874–81. doi: 10.1007/s12020-021-02631-2 (PMC8159788; doi:10.1007/s12020-021-02631-2)
Supplement: Supplementary file 1 — Supplementary Figures 1 [file 12020_2021_2631_MOESM1_ESM.docx]

Supplementary Figure **1**. Multivariable adjusted one-way ANOVA on ranks assessing differences concerning continuous total testosterone levels between pax intensity quartiles (count per day [CPD]).


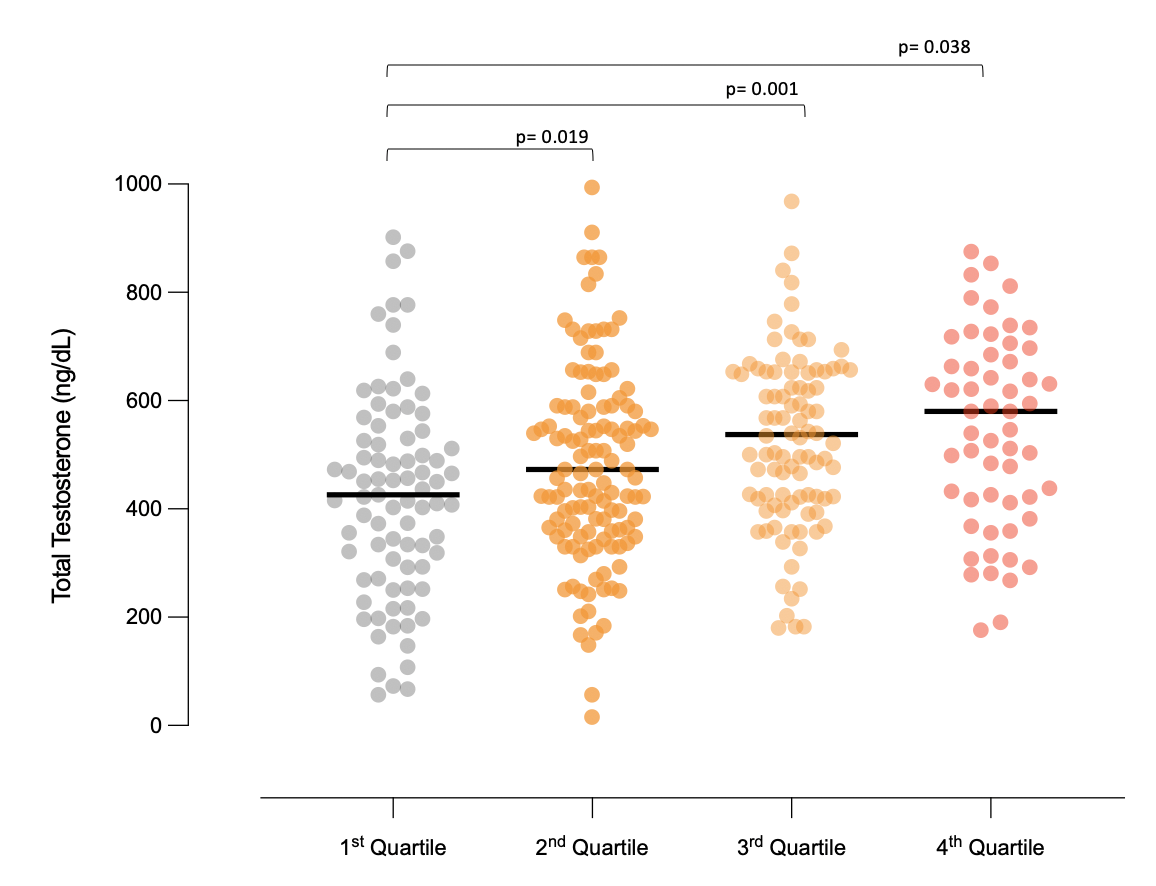


| **Pax Intensity quartiles** | **𝛽** | SE | 95%CI | | P value |
| --- | --- | --- | --- | --- | --- |
|  |  |  | Lower | Upper |  |
| *1^st^ quartile* | Ref. | **–** | **–** | **–** | **–** |
| *2^nd^ quartile* | 74,931 | 28,467 | 14,256 | 135,606 | **0.019** |
| *3^rd^ quartile* | 109,382 | 27,093 | 51,634 | 167,130 | **0.001** |
| *4^th^ quartile* | 82,470 | 36,187 | 5,338 | 159,602 | **0.038** |

Supplementary Figure **2.** Locally weighted scatter-plot smoother (LOWESS) function depicting multivariable adjusted predicted probability of impaired total testosterone (TT, ng/dl) according to total pax intensity (count per day [CPD]).


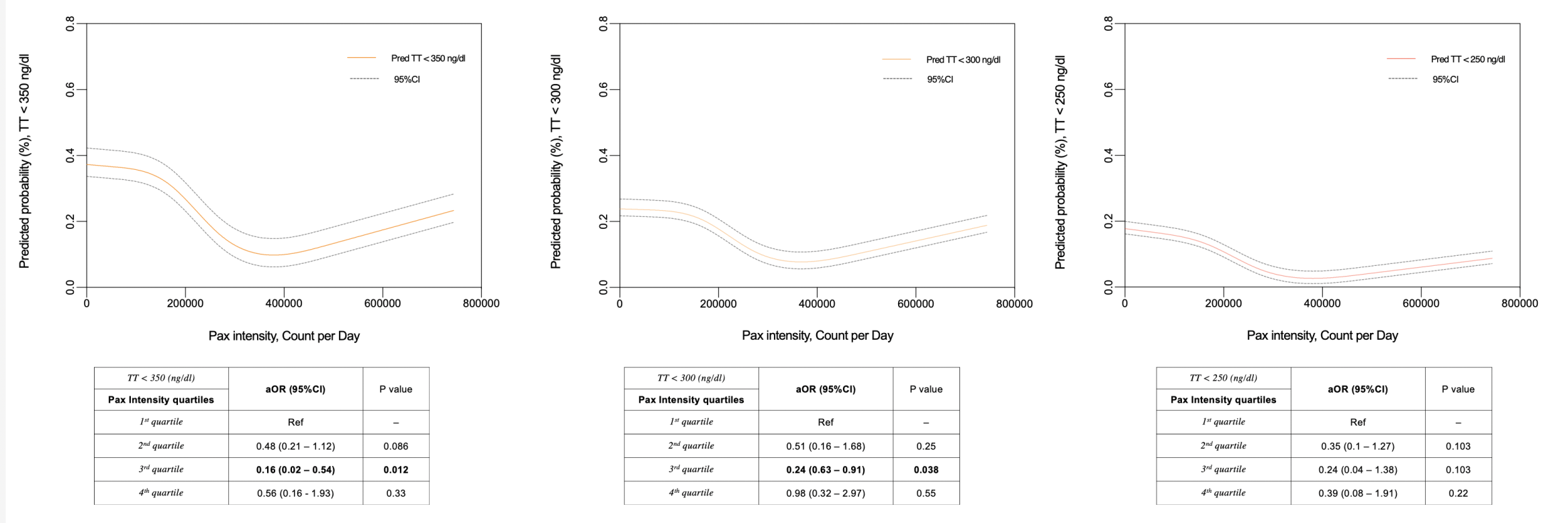


Supplementary Figure **3**. Locally weighted scatter-plot smoother (LOWESS) function depicting multivariable adjusted predicted probability of impaired free (a) and bioavailable (b) testosterone (fT, Bio-T, ng/dl) according to total pax intensity (count per day [CPD]).

**
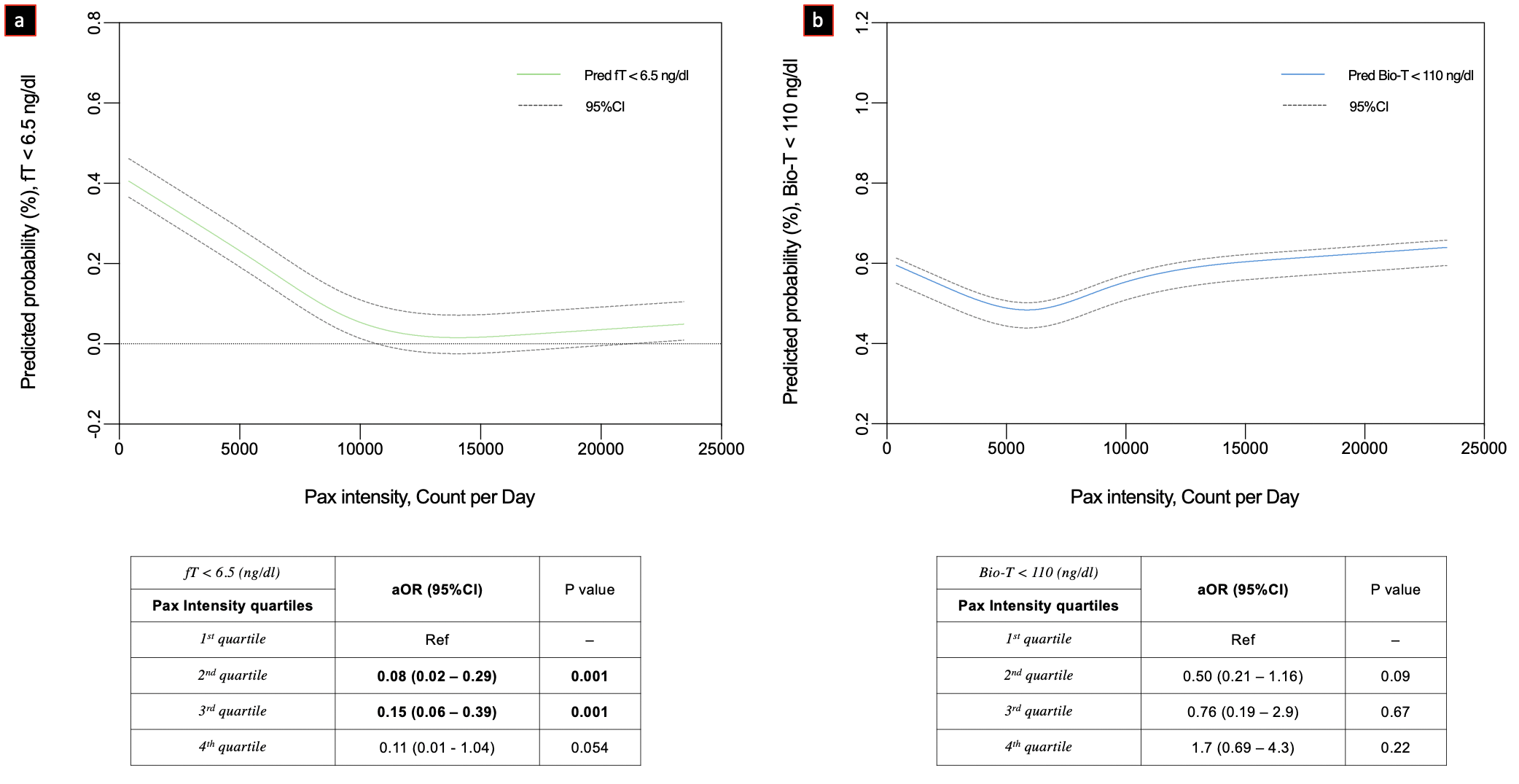
**
